# A scanning electron microscopic study evaluating the sealing ability of MTA, BiodentineTM, and new light-cure MTA used for furcal perforation repair 

**DOI:** 10.4317/jced.59755

**Published:** 2023-01-01

**Authors:** Pradnya Nagmode, Prachi Janbandhu, Abhishek Jagtap, Harshal Basatwar, Shubham Godge, Snehal Shinde

**Affiliations:** 1 Professor, Guide. Department of Conservative Dentistry & Endodontics, S.M.B.T Dental College, Amrutnagar, Ghulewadi, Sangamner, Dist: Ahmednagar, Maharashtra, India; 2 MDS Student. Department of Conservative Dentistry & Endodontics, S.M.B.T Dental College, Amrutnagar, Ghulewadi, Sangamner, Dist: Ahmednagar, Maharashtra, India; 3 MDS. Department of Conservative Dentistry & Endodontics, S.M.B.T Dental College, Amrutnagar, Ghulewadi, Sangamner, Dist: Ahmednagar, Maharashtra, India

## Abstract

**Background:**

To evaluate and compare the sealing ability of Mineral trioxide aggregate (MTA), BiodentineTM and light cure MTA used for the repair of furcal perforations using scanning electron microscope (SEM).

**Material and Methods:**

The study sample comprised 45 extracted mandibular molars. The teeth were embedded in modeling wax. Standard access cavities were prepared in each tooth using a round bur and non-end cutting bur with a high-speed handpiece with water spray. Furcal perforations were made on the centre of the pulpal floor of each tooth using a 0.5mm round bur. The teeth were then randomly divided into 3 experimental groups of 15 specimens each based on the materials used to seal the perforation; Group A: MTA; Group B: BiodentineTM; Group C: Light cure MTA. All the sealed perforations were compacted with a moist cotton pellet, and the samples were stored in a closed container for 24 hours to allow the repair materials to set completely. After 24 hours the samples were sectioned longitudinally and the extent of marginal adaptation was measured using scanning electron microscopy. The sealing ability was evaluated by measuring the gap (in microns) between the pulpal floor and the material used for the furcal repair.

**Results:**

The overall results showed that the marginal adaptation of light-cure MTA was better than both MTA as well as Biodentine. The mean space between the pulpal floor and the repair material was least for group C (2.29). Tuckey’s post hoc test showed that a significant difference (*p*<0.05) existed between group C and group A & B.

**Conclusions:**

Light-cure MTA exbibits good sealing ability to dentin when compared to conventionally used Biodentine and Mineral Trioxide Aggregate.

** Key words:**Calcium silicates, MTA, Biodentinte, New light-cure MTA, Perforation repair.

## Introduction

A perforation is a mechanical or pathologic communication between the external tooth surface and the root canal system which may be a result of pathologic factors such as caries or resorption, as well as iatrogenic factors (1). In multi-rooted teeth, periodontitis and irreversible attachment loss can result from furcal perforations of the pulpal floor (2).

Accidental perforations of the pulpal floor are a common endodontic mishap that might have a negative impact on the treatment outcome. In these cases, the prognosis is affected by a number of factors, including the location, size, and time of perforation. The ability of the material employed to seal the perforation is also an important factor that impacts the treatment prognosis (3).

Various materials have been employed to seal furcal perforations in the past such as amalgam, reinforced zinc oxide eugenol, super EBA, and calcium hydroxide. Glass ionomer cement, composite resins, MTA, biodentine, bioaggregate, platelet-rich fibrin (PRF), platelet-rich plasma (PRP) are used commonly currently. However, none of these materials possess all of the characteristics of an ideal repair material. An ideal perforation repair material should be biocompatible, dimensionally sTable, nontoxic, noncarcinogenic, and insoluble in bodily fluids (2). The material should also have the property to stimulate osteogenesis, and cementogenesis, and most importantly, have excellent sealing ability. Because of their excellent biocompatibility and ability to produce calcium-phosphate precipitation at the interface with periodontal tissue, calcium silicates have been the material of choice for sealing furcation perforations (3).

MTA has been the most extensively used perforation repair material, since being first discovered by Torabinejad at Loma Linda University in California, USA, in 1993. It is biocompatible, has good marginal adaptation, and has low cytotoxicity. MTA is a bioactive cement that can generate the ideal microenvironment for repair because of its tissue compatibility and antibacterial properties. It can stimulate the growth of periodontal ligament cells, osteoblast adhesion, and bone regeneration due to its cementogenic and osteogenic capabilities. When in contact with tissues, MTA activates bone markers necessary for biomineralization and the healing of periapical bone defects while also stimulating immune system cells to generate lymphokines that promote cementum repair and regeneration (4). It does, however, have a few disadvantages, which include the potential for discoloration, a long setting time (3 h), difficult handling, and a higher cost. MTA-Angelus (Angelus, Londrina, PR, Brazil) is a newer MTA formulation with a faster setting time (10 min) (2). For the purpose of overcoming the limitations of MTA, new calcium silicate-based bioactive cement was introduced in 2011 in the name of Biodentine.

Biodentine (Septodont, Saint-Maurdes-Fosses, France) has been used as an alternate for MTA since it has similar qualities to MTA but with the additional advantage of a faster setting time and better handling. It has a modified powder constituent, setting accelerators and softeners, and is available as a new predosed capsule formulation that contributes to the improvement of the physical properties of biodentine (5). Biodentine has been found to induce the healing of furcation perforations when in contact with periradicular tissues because it created a biomineralizing microenvironment with a minimal inflammatory reaction (4).

Cell viability and tissue healing are both known to be promoted by MTA and Biodentine. These calcium silicate-based bioceramics can also promote regenerative responses in natural tissues, such as osteoinduction, which is similar to that of hydroxyapatite (Raghavendra *et al*., 2017). Therefore, the effectiveness and outcome of endodontic applications, such as pulp capping, root-end filling, perforation repair, and pulp regeneration, were intrinsically linked to the biocompatibility and bioactivity of these calcium silicate-based bioceramics (6).

Incorporating light-curable resins has been proposed for many materials, such as the resin-modified glass-ionomer cement, to reduce their setting time as well as enhance their mechanical properties. MTA incorporated with light-curable resin will have a shorter setting time, extending its therapeutic use and allowing it to be used in wet and blood-contaminated surgical sites. 2 According to a 2010 study by Ricci Vivan *et al*. (7), light-cured MTA cement had lower solubility values (0.10 percent), as a result of its quick setting.

Given this, the aim of this study was to evaluate and compare the sealing ability of the most commonly used calcium silicate-based materials, MTA, and Biodentine, with the new light-cure MTA for the repair of furcation perforations using scanning electron microscopy (SEM). The primary hypothesis of this study was that there is no difference in the sealing ability of mineral trioxide aggregate, Biodentine, and light-cure mineral trioxide aggregate in the repair of furcation perforations.

## Material and Methods

Forty-five extracted mandibular molar teeth with mature root apices were selected and stored in 10% formalin solution until further use. The exclusion criteria included hypoplastic, carious, restored, and fractured teeth as well as teeth with root resorption where the furcal area could not be involved.

The samples were stored in 5.25% sodium hypochlorite for 24 hours to remove any tissue remnants after which the samples were washed and stored in saline until the tooth preparation was done. All the forty-five samples were then embedded in modeling wax.

Standard access cavities were prepared in each tooth using a round diamond bur and non-end cutting bur in a high-speed handpiece with water spray. A 0.5mm round bur was used to make furcation perforations on the center of the pulpal floor to standardize the size of the perforations.

After the furcal perforations were made, the blocks were randomly divided into 3 experimental groups based on the material used to seal the perforations: Group A (n=15); Group B (n=15); Group C(n=15):

Group A : MTA Group (Angelus, Londrina, PR, Brazil) The powder and liquid was dispensed on a glass slab and mixed in a circular motion, then carried using an MTA carrier to seal the perforation on the pulpal floor.

Group B : Biodentine Group (BiodentineTM, Septodont, France) The powder and liquid was mixed according to the manufacturer’s instructions and applied on the perforation site.

Group C : Light cure MTA (Dentigrate, Dentact Solutions Private Limited, India) The paste was filled into the perforation with the help of a bent needle provided by the manufacturer and cured for 1 minute as per the manufacturer’s instructions. Paste consists of a mixture of Calcium Oxide, Silicon Oxide, Bismuth Oxide, Methacrylate resins, photoinitiators and stabilizers.

The samples were stored in a closed container for 24 hours after compacting each sealed perforation site using a moist cotton pellet in order to allow the repair materials to be set completely. After 24 hours the samples were sectioned longitudinally the perforated sample of the teeth was taken for scanning electron microscopic examination.

The samples were gold-sputtered and viewed under SEM in different magnifications (200x, 500x, 1000x, 2000x, 3000x) for evaluating the sealing ability between the materials. The sealing ability was determined by measuring the gap (in microns) between the pulpal floor and the material used for sealing the perforation site.

-Statistical analysis

**Figure 1:**
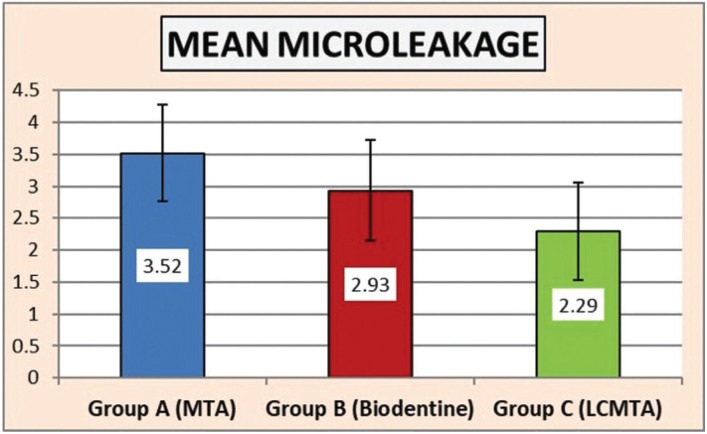
Descriptive statistics of microleakage (space in microns) between pulpal floor and repair material.

**Figure 2:**
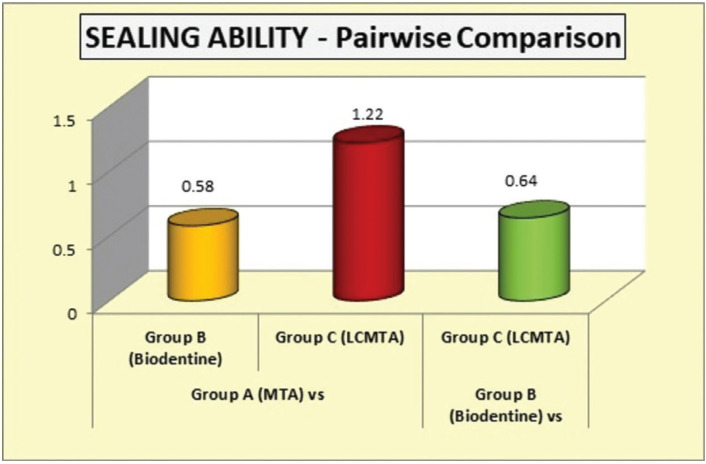
Comparative statistics of sealing ability (space in microns) between pulpal floor and repair material.

**Figure 3:**
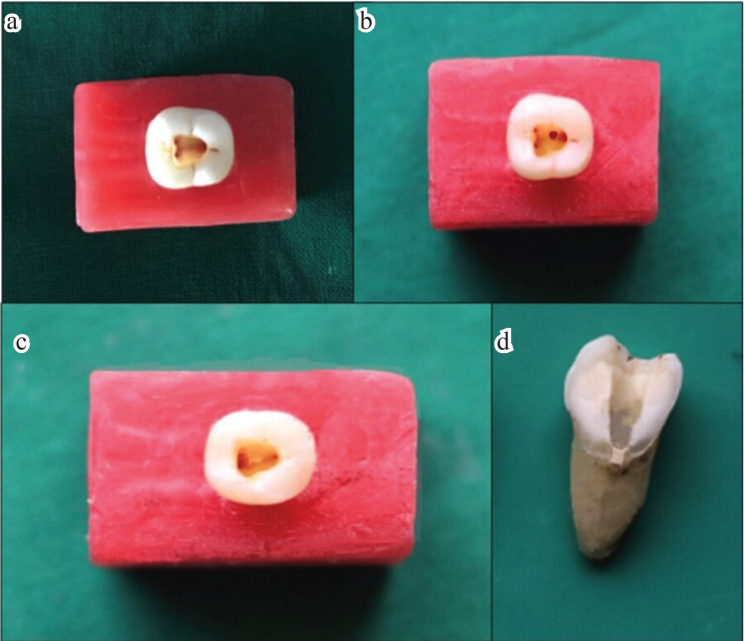
a) access cavity prepared b) standardized perforation in the centre of the pulpal floor c) sealed perforation d) longitudinal section.

**Figure 4:**
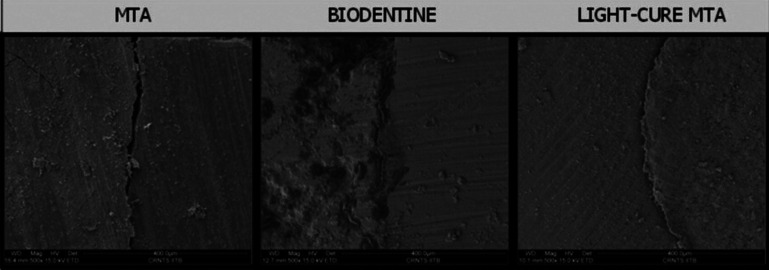
SEM images showing gap between pulpal floor and repair material.

Data analysis was carried out using SPSS software (SPSS version 18.0, SPSS, Chicago, IL, USA). One-way Anova ‘F’ test was used to compare the marginal adaptation of the three groups. Tukeys post hoc test was used for pairwise comparison (Figs. 1-4,  Table 1, Table 2).

**Table 1:**
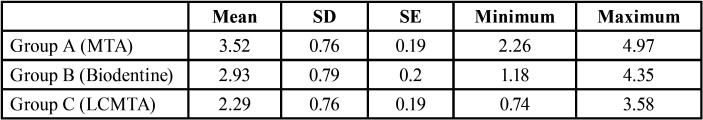
Descriptive statistics of sealing ability (space in microns) between pulpal floor and repair material.

**Table 2:**
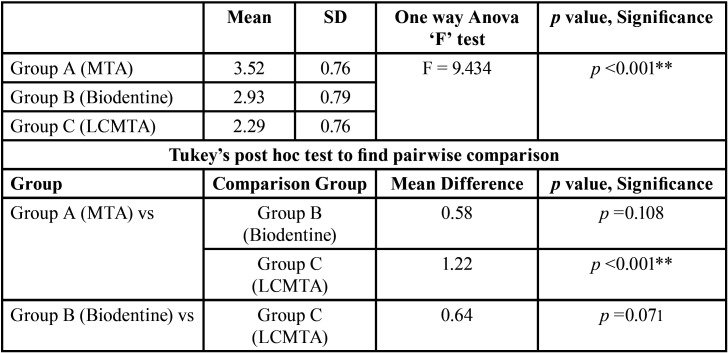
Comparative statistics of sealing ability (space in microns) between pulpal floor and repair material.

## Results

The lowest sealing ability assessed in terms of gap (in microns) was recorded for Group A (3.52±0.76, minimum value 2.26 µ, and maximum value 4.97 µ) that was followed by Group B (2.93±0.79, minimum value 1.18 µ, maximum value 4.35 µ) while the highest sealing ability was recorded for Group C (2.29±0.76, minimum value 0.74 µ, maximum value 3.58 µ).

One-way Anova ‘F’ test was used to compare the marginal adaptation of the three groups tested using the SPSS Statistical Package (SPSS Statistics for Windows, Version 17.0, SPSS Inc., Chicago, IL, USA). The ANOVA test revealed that there was a statistically significant difference in the mean gap (in microns) between at least two groups (*P*<0.05)

Tukeys post hoc test for pairwise comparison showed that Group C exhibited statistically significant (*P*<0.05) better sealing than Group A. There was no statistically significant difference between groups A and B and groups B and C.

## Discussion

The effective seal between the root canal system and the periodontal ligament is necessary for successful perforation repair. This seal depends on various properties of the repair material used such as marginal adaptation, adhesion, solubility, and volume changes in the cement used (5). For this reason, we compared the marginal adaptation of a newer calcium silicate-based cement i.e., Light-cured MTA with more commonly employed MTA-Angelus, and Biodentine in this study.

MTA’s biocompatibility and sealing abilities have been demonstrated in numerous studies (8). MTA’s cementogenic property is a result of its ability to release a large proportion of calcium ions, which interact with phosphate groups in the surrounding tissue fluid to form hydroxyapatite on its surface (9).

Ghanbari *et al*., (2008) examined the sealing ability of amalgam and MTA in repairing furcal perforations and concluded that MTA performed better than amalgam when used for perforation repair, especially when employed within a short period of the perforation, based on microscopic inspection (10).

When Lee SJ *et al*. (11) compared mineral trioxide aggregate with amalgam and Intermediate Restorative Material (IRM) for perforation repair, MTA showed significantly less leakage when compared to IRM and amalgam.

Compared to RetroMTA and ProRoot MTA used in furcation repair of mandibular molars, Sinkar *et al*., (2015) (12) found that Biodentine has superior sealing ability and the least microleakage. This study was conducted utilizing a dye extraction leakage method.

Guneser *et al*. theorized that Biodentine’s better interlocking with dentin may be due to its uniform components and overall smaller particle size when compared to MTA (13).

Biodentine’s adhesion to dentinal tubules could potentially be attributed to tag-like structures within the tubules that act as a micromechanical anchor (14). According to Han and Okiji (15), Biodentine had greater calcium and silicon ion uptake into dentin, resulting in the formation of tag-like structures, compared to MTA.

The results of this study revealed significant differences between the tested materials, with LC-MTA outperforming Biodentine and MTA-Angelus in terms of marginal adaption. As a result, the study’s null hypothesis was rejected.

The gap size between the dentin and the perforation repair material, as well as fluid leakage, gives a quantitative indication of the material’s sealing capability. Marginal adaptation of perforation repair materials evaluated using Scanning Electron Microscopy might therefore provide information about the repair materials’ sealing ability (2).

The findings of this study revealed that LC MTA had a greater sealing ability than Biodentine and MTA-Angelus, whereas Biodentine had a better sealing ability than MTA-Angelus. These findings are consistent with those of a prior study that used the dye penetration method and a Confocal Laser Scanning Microscope to assess the sealing ability of TheraCal (a light cure MTA product) and MTA with biodentine. They found that TheraCal LC exhibits less microleakage than other materials tested 7

Dentigrate Light-cure MTA, the calcium silicate-based material employed in this study, is a single-paste calcium silicate-based material recommended by the manufacturer for use as a pulp capping, perforation repair material, as well as a cavity liner under all filling materials. The polymerizable component’s setting reaction is light-activated. The improved sealing ability could be attributed to the material’s flow, which allows for quicker application and immediate setting. The tiny size of Biodentine particles, which facilitates adaptation at the cavity surface and filling contact, may explain why Biodentine has a better adaption property than MTA (2).

A possible explanation for the good marginal adaptation of Light cure MTA despite expected polymerization shrinkage is that this calcium silicate based material stimulates formation of hydroxyapatite onto the surface and provides a biological seal. It might resemble or function as a scaffold to facilitate dentin formation. It absorbs dentinal fluids, releasing calcium and hydroxide ions as a result. Apatite formation on the undersurface of the MTA, being one of the tooth’s immediate responses, enhances the product’s natural sealing ability. However further research regarding this apatite stimulating and calcium release ability is warranted.

A limitation of this study is that sectioning of the tooth through the perforation site might lead to development of cracks, fissures and also cause material drag to appear which may impact the apparent marginal adaptation of the repair material assessed through scanning electron microscopy. However, this methodology is commonly employed in many studies testing sealing ability through assessment of marginal adaptation of repair materials. Alternate methodologies that test the sealing ability include dye penetration, bacterial leakage, protein leakage and computerized fluid filtration assessment may be used for future assessments.

Due to a paucity of long-term observational studies, determining which material among MTA, Biodentine, and light-cure MTA is preferable is difficult. Biodentine has a lower wash-out resistance and has an unfavorable radiopacity compared to MTA. However, Biodentine is preferred due to its ease of use and cost. LC-MTA has excellent sealing properties, however, it needs to be tested further to determine its long-term biological and clinical efficacy.

## Conclusions

When compared to conventionally used Biodentine and Mineral Trioxide Aggregate, Dentigrate Light-cure MTA demonstrates a higher sealing ability to dentin. Materials with novel compositions, on the other hand, should be thoroughly investigated before being used in clinical settings. To determine its biological and clinical efficacy, further research on biocompatibility, solubility, calcium release properties, and remineralizing potential is required. Before Dentigrate Light-cure MTA can be employed as a perforation repair material, more in-vitro and in-vivo study is warranted.
